# Association between household second-hand smoke and low birth weight in sub-Saharan Africa

**DOI:** 10.1371/journal.pone.0330214

**Published:** 2025-08-21

**Authors:** Sanni Yaya, Emmanuel Kolawole Odusina

**Affiliations:** 1 The George Institute for Global Health, Imperial College London, London, United Kingdom; 2 Department of Demography and Social Statistics, Federal University, Oye Ekiti, Ekiti State, Nigeria; African Population and Health Research Center, KENYA

## Abstract

**Background:**

The reduction of maternal and child morbidity and mortality is essential for achieving the Sustainable Development Goals (especially goal 3 – wellbeing of mothers and children) in sub-Saharan Africa (SSA). Exposure to household second-hand smoke (SHS) has been linked to adverse birth outcomes, yet limited evidence exists on its impact on low birth weight (LBW) in SSA. This study examines the association between household SHS exposure and LBW across ten SSA countries using recent national survey data.

**Methods:**

A cross-sectional study was conducted using data from the Demographic and Health Surveys (DHS) from ten SSA countries collected between 2020 and 2024. The sample included 45,684 women aged 15–49 who had given birth in the five years preceding the surveys. The primary outcome variable was LBW, defined as birth weight <2500 grams. SHS exposure was determined based on household smoking behavior. Bivariate analysis was conducted using Chi-square tests, and multivariate logistic regression models were employed to estimate adjusted odds ratios (AOR) with 95% confidence intervals (CI), controlling for maternal, household, and reproductive health factors.

**Results:**

Overall, 6.3% of women reported exposure to SHS, and 11.9% of births were classified as LBW. After adjusting for potential confounders, SHS exposure was significantly associated with increased odds of LBW (AOR = 1.28; 95% CI: 1.12–1.46, p < 0.01). The association was particularly pronounced in urban areas (AOR = 1.39; 95% CI: 1.17–1.65, p < 0.01). Other significant predictors of LBW included maternal age < 20 years, lower educational attainment, low antenatal care (ANC) attendance, and socioeconomic status.

**Conclusion:**

Household SHS exposure is an independent risk factor for LBW in SSA. Given the significant burden of LBW on neonatal health, policies targeting SHS reduction—such as household smoking bans and integrating SHS awareness into prenatal care—should be prioritized. Future longitudinal studies should explore causal mechanisms in greater detail.

## Background

Second-hand smoke (SHS) exposure is a critical global public health issue, significantly affecting maternal and child health. SHS is a combination of side stream smoke from burning tobacco products and mainstream smoke exhaled by a smoker, containing over 7,000 chemicals, including formaldehyde, benzene, carbon monoxide, and hydrogen cyanide, of which at least 69 are known carcinogens [1]. Exposure to these toxic substances during pregnancy is linked to placental insufficiency, fetal hypoxia, intrauterine growth restriction (IUGR), and adverse birth outcomes, such as stillbirth, preterm birth, and low birth weight (LBW) [2,3]. These effects primarily occur due to vasoconstriction, impaired placental blood flow, and disruption of normal fetal growth, leading to long-term health complications [4].

Globally, SHS exposure is responsible for approximately 600,000 deaths annually, disproportionately affecting pregnant women and children [5]. The World Health Organization (WHO) estimates that 35% of women and 40% of children worldwide are exposed to SHS, primarily within their own homes [6,7]. Women and children are at particularly high risk due to prolonged indoor exposure, sociocultural norms that limit women’s ability to enforce smoke-free environments, and poor ventilation in many households [8]. While SHS-related health risks are well-documented in high-income countries (HICs), research on its impact in low- and middle-income countries (LMICs), particularly in sub-Saharan Africa (SSA), remains limited [9].

Unlike many HICs, where strict tobacco control policies—such as smoking bans in indoor spaces, taxation, and public awareness campaigns—have significantly reduced SHS exposure, SSA countries face weak enforcement and inadequate regulations. The Global Adult Tobacco Survey (GATS) indicates that in some SSA countries, 20–40% of non-smoking women are regularly exposed to SHS at home due to household smoking by male family members [10]. The lack of public health awareness, limited smoking restrictions, and sociocultural norms that tolerate indoor smoking further increase exposure risks [11].

Additionally, urbanization and shifting smoking patterns have contributed to higher SHS exposure rates in SSA. While smoking prevalence has traditionally been lower in SSA than in other regions, increasing tobacco marketing, rising disposable incomes, and weak anti-smoking policies have contributed to a growing number of young men smoking indoors. The availability of cheap, locally produced cigarettes further exacerbates this issue, increasing SHS exposure risks for women and children [12].

LBW, defined as a birth weight below 2,500 grams, is a key predictor of neonatal morbidity, mortality, and long-term health complications. LBW infants face higher risks of infections, respiratory distress, hypothermia, poor immune function, and developmental delays [13]. In the long term, LBW is associated with increased risks of hypertension, cardiovascular disorders, and type 2 diabetes in adulthood [14]. The WHO estimates that 15–20% of all newborns worldwide are born with LBW, with the highest prevalence occurring in LMICs, including (particularly in) SSA [15,16].

The relationship between maternal SHS exposure and LBW has been extensively studied in North America, Europe, and Asia, with research showing that infants of SHS-exposed mothers are 25–30% more likely to have LBW compared to non-exposed mothers. However, limited research has explored this association in SSA, where the socioeconomic, environmental, and health system factors differ significantly from other regions. Given the already high neonatal and infant mortality rates in SSA, identifying modifiable risk factors such as SHS exposure is essential for improving maternal and child health outcomes [17].

Despite the well-documented link between SHS and adverse birth outcomes in other regions, research on SHS and LBW in SSA remains scarce. Some country-specific studies suggest a strong association between household SHS exposure and adverse birth outcomes. For example, a study in Malaysia found that increase in the exposure to the smoke of one cigarette decrease birth weight by 12.9 g.[18], while a study in China reported significantly lower birth weights among infants born to SHS-exposed mothers [11,19]. These findings suggest that household tobacco smoke exposure may be an underappreciated contributor to poor birth outcomes in SSA, necessitating more region-specific research and policy interventions.

Given SSA’s high maternal and child mortality rates and the limited research on SHS exposure in the region, this study aims to analyze the association between SHS exposure and LBW using nationally representative Demographic and Health Survey (DHS) data from ten SSA countries collected between 2020 and 2024. By addressing this research gap, the study will provide critical insights for policymakers and public health officials to develop interventions aimed at reducing SHS exposure in SSA households. These efforts align with Sustainable Development Goal (SDG) 3, which seeks to reduce neonatal mortality and promote overall health and well-being.

## Methods

### Study design and data source

This study utilized the most recent Demographic and Health Surveys (DHS) data, a nationally representative dataset providing comprehensive information on maternal and child health indicators. Data from ten sub-Saharan African (SSA) countries were analyzed, focusing on surveys conducted between 2020 and 2024 to ensure relevance and minimize recall bias among respondents. By selecting only, the most recent surveys conducted within the last four years, this study aimed to reduce potential errors associated with maternal memory lapse in birth weight reporting.

### Study population and sample size

Eligible respondents were women of reproductive age (15–49 years) who had given birth within the five years preceding the survey. The total sample size comprised 45,684 women, making this one of the largest regional studies examining the relationship between SHS exposure and low birth weight (LBW) in SSA.

### Outcome variable

The primary outcome variable, LBW, was defined as a birth weight of less than 2,500 grams. To facilitate analysis, LBW was dichotomized into two categories: LBW (birth weight below 2,500 grams) and normal birth weight (birth weight of 2,500 grams or more).

### Exposure variable

The independent variable, household second-hand smoke (SHS) exposure, was assessed based on self-reported smoking behaviors within the household. Respondents were categorized as exposed if they reported that at least one household member smokes regularly inside the home, while those who reported no smoking behavior within the household were classified as non-exposed.

### Covariates and potential confounders

To account for confounding factors that could influence birth weight, the analysis controlled for a range of maternal, household, and reproductive characteristics. Maternal factors included age [15–48], educational level ((no formal education), (primary (basic literacy education), secondary (high school level), and post-secondary (college/university education), employment status (working or not working), and prenatal care attendance (<8 or >8) [20,21]. Household characteristics included wealth index, as used and defined by the DHS program (poorest, poorer, middle, richer and richest), type of cooking fuel used (“clean” fuels refer to electricity, LPG, natural gas, and biogas, while “pollutant” fuels include biomass (wood, dung), charcoal, and kerosene), and place of residence (urban or rural). Reproductive history was also considered, including parity (≥5 children, 3–4 children and 1–2 children) and maternal age at first birth (<20, 20–29 and >29 years). These variables were selected based on prior research indicating their influence on fetal growth and birth weight outcomes [2–4,6,20].

### Ethical considerations

Ethical clearance was not required since the researchers did not engage directly with respondents. The study utilized data obtained from the MEASURE DHS Program, with access granted through an online request submitted via the DHS Program website.

## Results

### Prevalence of Second-hand Smoke (SHS) Exposure and Low Birth Weight (LBW)

Table 1 presents the prevalence of SHS exposure and LBW among respondents across the ten selected sub-Saharan African countries. Among the total sample of 45,684 women, 6.3% reported being exposed to SHS in their household environment, while 11.9% had infants with LBW. The prevalence of SHS exposure varied significantly across countries. Gabon recorded the highest prevalence of household SHS exposure at 35.7%, followed by Madagascar at 10.9%. In contrast, Gambia had the lowest prevalence at 0.3%, indicating wide disparities in household smoking behaviors across SSA. In terms of LBW, Mauritania had the highest prevalence at 24.6%, followed by Côte d’Ivoire (13.1%), Mozambique (13.3%), and Gabon (14.1%). The lowest LBW prevalence was recorded in Kenya (8.5%) and Tanzania (8.5%), suggesting that country-specific factors, such as maternal nutrition, healthcare access, and environmental exposures, may influence birth weight outcomes.

**Table 1:** Data distribution among women aged 15-49 years in selected sub-Saharan African countries.

Country	Survey Year	n = 45684	Prevalence of SHS (%)	Prevalence of LBW (%)
Burkina Faso	2021	5716	2.1	12.0
Cote d’Ivoire	2021	4577	1.4	13.1
Gabon	2019/2021	5473	35.7	14.1
Gambia	2019/2020	5853	0.3	9.5
Ghana	2022	4189	0.8	11.0
Kenya	2022	4707	0.8	8.5
Madagascar	2021	4747	10.9	12.8
Mauritania	2019-2021	1850	3.4	24.6
Mozambique	2022/2023	3391	1.4	13.3
Tanzania	2022	5182	0.5	8.5
Total		45684	6.3	11.8

### Association between SHS exposure and LBW

Table 2 examines the percentage distribution of respondents by SHS exposure across key maternal, socioeconomic, new born profiles and reproductive characteristics. The results indicated that infants with LBW were more among mothers exposed to SHS. Specifically, new born profiles revealed that, of 6.3% of children that were exposed to secondhand smoke, 16.0% of mothers with LBW infants reported household SHS exposure, compared to 11.6% of mothers with no exposure to SHS. In addition, of the total number of children exposed to secondhand smoke, 19.1% of them had smaller than average and very small size at birth.

**Table 2:** Selected characteristics of respondents by SHS.

Characteristics	All (n = 45684) n(%)*	Second hand smoke exposure		P-value
		Not exposed (n = 42803) n (%)*	Exposed (n = 2881) n (%)*	
SHS				
Not exposed	42803 (93.7)	–	–	
Exposed	2881 (6.3)	–	–	
CBW				
NBW	40262 (88.1)	37842 (88.4)	2420 (84.0)	
LBW	5422 (11.9)	4961 (11.6)	461 (16.0)	<0.001
Maternal age				
15-19	3266 (7.2)	3110 (7.3)	155 (5.4)	
20-24	10943 (24.0)	10296 (24.1)	647 (22.5)	
25-29	12001 (26.3)	11258 (26.3)	743 (25.8)	
30-34	9553 (20.9)	8940 (20.9)	612 (21.3)	
35-39	6566 (14.4)	6102 (14.3)	464 (16.1)	
40-44	2732 (6.0)	2521 (5.9)	211 (7.3)	
45-49	623 (1.4)	574 (1.3)	49 (1.7)	<0.001
Maternal age at first birth (years)				
<20	24213 (53.0)	22600 (52.8)	1614 (56.0)	
20-29	20233 (44.3)	19059 (44.5)	1175 (40.8)	
>29	1237 (2.7)	1144 (2.7)	93 (3.2)	<0.001
Marital Status				
Never married	4369 (9.6)	3851 (9.0)	518 [18]	
Married	38274 (83.8)	36242 (84.7)	2032 (70.5)	
Widow/Separated/Divorced	3042 (6.7)	2710 (6.3)	332 (11.5)	<0.001
Maternal education level				
No formal education	12618 (27.6)	12225 (28.6)	393 (13.6)	
Primary	12811 (28.0)	12074 (28.2)	738 (25.6)	
Secondary	16978 (37.2)	15528 (36.3)	1449 (50.3)	
Post secondary	3277 (7.17)	2976 (7.0)	302 (10.5)	<0.001
Maternal occupation				
Not working	17463 (38.3)	16230 (38.0)	1233 (42.8)	
Working	28173 (61.7)	26528 (62.0)	1645 (57.2)	<0.001
Residence				
Urban	22824 (50.0)	20730 (48.4)	2093 (72.7)	
Rural	22861 (50.0)	22072 (51.6)	788 (27.4)	
Wealth index				
Poorest	7481 (16.4)	6878 (16.1)	602 (20.9)	
Poorer	8780 (19.2)	8128 (19.0)	651 (22.6)	
Middle	9328 (20.4)	8739 (20.4)	588 (20.4)	
Richer	10204 (22.3)	9645 (22.5)	560 (19.4)	
Richest	9891 (21.7)	9412 (22.0)	480 (16.6)	<0.001
Parity				
≥5	10684 (23.3)	9878 (23.1)	805 (28.0)	
3-4	14552 (31.9)	13666 (31.9)	886 (30.7)	
1-2	20448 (44.7)	19258 (45.0)	1190 (41.3)	<0.001
ANC				
<8 visits	35672 (91.3)	33722 (91.7)	1951 (89.0)	
8 + visits	3305 (8.5)	3063 (8.3)	242 (11.0)	0.850
Birth interval				
<33 months	4575 (77.1)	4103 (77.5)	471 (73.6)	
33 + months	1361 (22.9)	1192 (22.5)	169 (26.4)	0.006
Child’s size at birth				
Very large	4969 (10.9)	4591(10.7)	378 (13.1)	
Larger than average	10074 (22.1)	9547 (22.3)	527 (18.3)	
Average	24547 (53.7)	23126 (54.0)	1422(49.3)	
Smaller than average	4021 (8.8)	3680 (8.6)	341 (11.8)	
Very small	1959 (4.3)	1748 (4.1)	210 (7.3)	
Don’t know	114 (0.25)	111 (0.3)	4 (0,1)	<0.001
Cooking Fuel				
Clean	16275 (35.6)	14217 (33.2)	2058 (71.4)	
Pollutant	29409 (64.4)	28585 (66.7)	824 (38.6)	<0.001

*Wealth index quintiles as used by the DHS program.

*Educational levels—primary (basic literacy education), secondary (high school level), and post-secondary (college/university education)

*“Clean” fuels refer to electricity, LPG, natural gas, and biogas, while “pollutant” fuels include biomass (wood, dung), charcoal, and kerosene.

Further analysis of maternal characteristics revealed that women exposed to SHS were aged 30–49 years, had their first birth either before 20 years or after 29 years, be unmarried, and have at least secondary education. Additionally, more of SHS-exposed women were unemployed, reside in urban areas, and disproportionately concentrated in poorer and poorest wealth index categories. They also had higher parity (five or more children), attended at least eight antenatal care (ANC) visits, and use clean cooking fuel. These characteristics suggest that SHS exposure is more common among women in urban, low-income settings where household smoking is prevalent.

### Multivariable Analysis: Adjusted Odds Ratios (AOR) for SHS Exposure and LBW

Table 3 presents the results of the logistic regression analysis assessing the relationship between SHS exposure and LBW, adjusting for maternal, household, and reproductive factors. The findings indicate a statistically significant association between SHS exposure and LBW, even after controlling for potential confounders.

**Table 3:** Relationship between SHS exposure and low birth weight among selected sub-Saharan African countries.

SHS exposure	Low birth weight		P-value
	OR (95% CI)	AOR (95% CI)^2^	
Not exposed	1	1	
Exposed	1.41 (1.27-1.57)*	1.28 (1.12-1.46)*	<0.001

AOR: adjusted for maternal age, education, marital status, residence, wealth status, antenatal visits, cooking fuel, parity and occupation. *p < 0.001.

Compared to mothers who were not exposed to SHS, those who reported household SHS exposure had 1.28 times higher odds of delivering an LBW infant (AOR = 1.28; 95% CI: 1.12–1.46, p < 0.01).

### Urban-Rural Disparities in the SHS-LBW Relationship

Given that urban households may have different smoking behaviors and indoor air quality compared to rural settings, a separate analysis was conducted to examine whether the association between SHS exposure and LBW varied by place of residence. The results indicate that the risk of LBW was significantly higher among urban-dwelling women exposed to SHS than among their rural counterparts.

Compared to non-exposed mothers residing in urban areas, urban mothers exposed to SHS had 1.39 times higher odds of delivering an LBW infant (Table 4) (AOR = 1.39; 95% CI: 1.17–1.65, p < 0.01).

**Table 4:** Relationship between SHS exposure and low birth weight in urban-rural areas among selected sub-Saharan African countries.

	Rural Areas			Urban Areas		
SHS exposure	Low birth weight			Low birth weight		
	OR (95% CI)	AOR (95% CI)	P-value	OR (95% CI)	OR (95% CI)	P-value
Not exposed	1	1		1	1	
Exposed	1.14(0.96-1.37)	1.11 (0.89-1.37)	>0.005	1.58 (1.39-1.80)*	1.39 (1.17-1.65)*	<0.001

AOR: adjusted for maternal age, education, marital status, residence, wealth status, antenatal visits, cooking fuel, parity and occupation. *p < 0.001.

## Discussion

This study examined the association between household second-hand smoke (SHS) exposure and low birth weight (LBW) in ten sub-Saharan African (SSA) countries using nationally representative recent Demographic and Health Survey (DHS) data collected between 2020 and 2024. The findings indicate that 6.3% of the total sample reported exposure to SHS in their household, while 11.9% had infants classified as having LBW. The prevalence of SHS exposure varied significantly across countries, with Gabon (35.7%) and Madagascar (10.9%) recording the highest rates, while the lowest exposure was observed in Gambia (0.3%) and Kenya (0.8%). Similarly, LBW prevalence was highest in Mauritania (24.6%), followed by Gabon (14.1%), Côte d’Ivoire (13.1%), Mozambique (13.3%), and Madagascar (12.8%), underscoring substantial regional disparities in birth weight outcomes. High prevalence of LBW in Mauritania was not unconnected with factors such as limited access to quality antenatal care and high maternal malnutrition rates.

A key finding of the study is the strong and statistically significant association between SHS exposure and LBW. Infants born to mothers exposed to SHS had a 28% increased likelihood of being born with LBW compared to infants of non-exposed mothers, even after adjusting for potential confounders such as maternal age, education, wealth index, parity, and antenatal care visits. This finding is consistent with previous research conducted in high-income countries, such as the United States and Greece, which also found elevated risks of LBW among SHS-exposed pregnant women [3,22,23]. The biological plausibility of this association is well-established; SHS exposure increases maternal carbon monoxide and nicotine intake, leading to placental vasoconstriction, fetal hypoxia, and impaired nutrient transfer, all of which contribute to intrauterine growth restriction (IUGR) and LBW [24].

The observed findings align with other studies identifying household SHS exposure as a significant risk factor for LBW and other adverse birth outcomes. Studies from North America, Europe, and Asia have consistently demonstrated that maternal exposure to SHS is associated with a higher likelihood of delivering an LBW infant. A meta-analysis by Leonardi-Bee et al. concluded that non-smoking pregnant women exposed to SHS had a 22% increased risk of LBW compared to unexposed mothers [25]. Similarly, a study conducted in China found that maternal SHS exposure resulted in a 27% higher probability of LBW [19]. Another investigation in Turkey identified a strong dose-response relationship, where the risk of LBW increased with both the frequency and duration of SHS exposure during pregnancy [26].

Findings from this study are also consistent with evidence from other low- and middle-income countries (LMICs), where SHS exposure remains a critical yet underexamined determinant of adverse birth outcomes. Research from India found that maternal SHS exposure was associated with a 30% higher risk of LBW, particularly among women living in multi-generational households where indoor smoking was common [27]. A study from Indonesia reported that pregnant women exposed to SHS at home had significantly lower mean birth weights than their unexposed counterparts [20]. Additionally, an analysis from Brazil emphasized that women from lower socioeconomic backgrounds were more likely to experience SHS exposure and consequently had a higher risk of delivering an LBW infant [12].

In SSA, limited research has focused specifically on the relationship between SHS exposure and LBW, despite the region’s high burden of maternal and neonatal mortality. However, studies conducted in low-income and middle-income countries have reported similar associations [28]. A Nigerian study found that improvement in socio-economic indices, maternal nutrition, health education, quality and quantity of the antenatal care services were important to reduce the menace of7.3% LBW [29]. In Ethiopia, researchers identified a significant inverse relationship between SHS exposure and birth weight, with infants of exposed mothers weighing significantly less than those of non-exposed mothers [30]. These studies reinforce the findings of this research, highlighting the urgent need for more targeted public health interventions addressing household SHS exposure in SSA.

One particularly noteworthy finding from this study is the evidence of urban-rural disparities in the SHS-LBW relationship. The adjusted odds ratios indicate that urban women exposed to SHS had a 39% higher likelihood of delivering an LBW infant compared to their rural counterparts. This suggests that SHS exposure may have a more pronounced effect in urban environments, possibly due to higher indoor smoking rates, reduced household ventilation, and greater cumulative exposure to environmental pollutants [31]. These findings underscore the need for targeted anti-smoking interventions in urban areas, where the concentration of smokers and limited household space may exacerbate SHS exposure risks. Though this finding is not consistent with the one conducted in Malaysia [32]. This finding is corroborated by a meta-analysis study from Asia and African continents [33].

Additionally, the study’s findings highlight the socioeconomic factors influencing SHS exposure and LBW outcomes. This result confirms that household SHS exposure is an independent risk factor for LBW, even when adjusting for other socioeconomic and maternal health variables. Additionally, other maternal factors were found to significantly influence birth weight outcomes. Women who were younger than 20 years or older than 35 years at first birth, had fewer ANC visits, or lived in poorer households were at higher risk of delivering an LBW infant. Notably, even after adjusting for these factors, SHS exposure remained a strong predictor of LBW, reinforcing the public health significance of reducing tobacco smoke exposure in household settings

Women with lower education levels, limited access to antenatal care, and those from poorer households were disproportionately affected by SHS exposure, exacerbating existing health inequalities. This is in line with global evidence suggesting that tobacco smoke exposure disproportionately impacts socioeconomically disadvantaged populations, reinforcing the need for policies targeting vulnerable groups [34]. Given that LBW is a leading cause of neonatal morbidity and mortality, addressing SHS exposure through targeted tobacco control measures could significantly improve birth outcomes in SSA [35].

This study has several implications for public health policy and intervention programs in SSA. Given the significant burden of LBW on neonatal health, implementing stricter household smoking bans and integrating SHS awareness campaigns into maternal healthcare services are essential steps toward mitigating the adverse effects of SHS exposure. Countries with high SHS prevalence, such as Gabon and Madagascar, should prioritize targeted anti-smoking interventions, particularly in urban areas where exposure appears to be more pronounced [36]. Strengthening tobacco control policies, promoting smoke-free environments, and increasing public awareness about the risks of SHS exposure could contribute to reducing the incidence of LBW in SSA [4].

Further research is needed to assess causal pathways, objective exposure measurements, and potential interventions that can effectively reduce SHS-related risks among pregnant women. Longitudinal studies should explore the cumulative effects of SHS exposure on fetal development, while intervention studies should evaluate the efficacy of community-based smoke-free policies in SSA [5]. Addressing SHS exposure should be an integral part of maternal and child health programs to reduce the burden of LBW and associated complications [1]. Public health policies should focus on education, policy enforcement, and community-based awareness programs to mitigate the impact of SHS exposure on birth outcomes in SSA [2].

### Strengths and Limitations of the Study

This study has several strengths. First, it utilizes nationally representative DHS data from ten sub-Saharan African countries, enhancing the generalizability of the findings. The large sample size of over 45,000 women provides robust statistical power to detect significant associations. Additionally, the use of multivariable logistic regression allows for controlling various confounders, improving the accuracy of estimates. Moreover, the study contributes to a relatively underexplored research area in SSA by providing evidence on the relationship between SHS exposure and LBW in the region.

Despite its strengths, this study has some limitations. The reliance on self-reported SHS exposure may introduce recall and social desirability bias, potentially leading to misclassification. Additionally, DHS data do not capture objective biomarkers of SHS exposure, which would strengthen causal inference. The cross-sectional nature of the study limits the ability to establish temporal causation between SHS exposure and LBW. Future research should consider potential factors responsible for high SHS and LBW especially in Gabon and Mauritania respectively. Furthermore, potential unmeasured confounders, such as maternal nutrition and indoor air pollution from other sources, may influence the observed associations. Future research should incorporate longitudinal designs and objective exposure assessments to address these limitations.

## Conclusion

This study provides compelling evidence that household second-hand smoke (SHS) exposure is a significant risk factor for low birth weight (LBW) in sub-Saharan Africa (SSA). The findings underscore the urgent need for comprehensive public health interventions to mitigate the adverse effects of SHS exposure on maternal and neonatal health. The strong association between SHS exposure and LBW, particularly in urban settings, highlights the necessity of targeted anti-smoking policies, increased public awareness, and stricter enforcement of smoke-free household regulations.

Given the high burden of neonatal morbidity and mortality associated with LBW, governments and policymakers should prioritize SHS reduction strategies, including community-based educational programs, stricter tobacco control regulations, and improved maternal healthcare services that integrate SHS awareness campaigns. Addressing SHS exposure as a preventable risk factor is crucial to improving birth outcomes and achieving Sustainable Development Goal 3, which aims to ensure healthy lives and promote well-being for all.

Future research should focus on longitudinal studies that explore the causal mechanisms linking SHS exposure to LBW while incorporating objective biomarkers to strengthen causal inference. Additionally, intervention studies should assess the effectiveness of smoke-free policies and educational campaigns in reducing SHS exposure among pregnant women. By implementing evidence-based strategies, SSA can make significant strides toward reducing the incidence of LBW. Future research should consider potential factors responsible for high SHS and LBW especially in Gabon and Mauritania respectively and improving neonatal health outcomes across the region.

## Supporting information

S1 Table Selected characteristics of respondents by LBW. (DOCX)
