# Supplementary material for: Association between household second-hand smoke and low birth weight in sub-Saharan Africa
Source: PLoS One. 2025 Aug 21;20(8):e0330214. doi: 10.1371/journal.pone.0330214 (PMC12370086; doi:10.1371/journal.pone.0330214)
Supplement: S1 Table — (DOCX) [file pone.0330214.s001.docx]

**S1 Table.** Selected characteristics of respondents by LBW

| Characteristics | All (n= 45684)  n(%)* | Urban Area (n=22824)  n(%)* | Rural Area (n=22860)  n(%)* |
| --- | --- | --- | --- |
|  |  |  |  |
| SHS |  |  |  |
| Not exposed | 42803 (93.7) | 20730 (90.8) | 22072 (96.5) |
| Exposed | 2881 (6.3) | 2093 (9.2) | 788 (3.5) |
| CBW |  |  |  |
| NBW | 40262 (88.1) | 20039 (87.8) | 20223 (88.5) |
| LBW | 5422 (11.9) | 2784 (12.2) | 2638 (11.5) |
| Maternal age |  |  |  |
| 15-19 | 3266 (7.2) | 1272 (5.6) | 1994 (8.7) |
| 20-24 | 10943 (24.0) | 4896 (21.5) | 6047 (26.5) |
| 25-29 | 12001 (26.3) | 6366(27.9) | 5636 (24.7) |
| 30-34 | 9553 (20.9) | 5147 (22.6) | 4405 (19.3) |
| 35-39 | 6566 (14.4) | 3510 (15.4) | 3056 (13.4) |
| 40-44 | 2732 (6.0) | 1374 (6.0) | 1358 (5.9) |
| 45-49 | 623 (1.4) | 258 (1.1) | 365 (1.6) |
|  |  |  |  |
| Maternal age at first birth (years) |  |  |  |
| <20 | 24213 (53.0) | 10457 (45.8) | 13757 (60.2) |
| 20-29 | 20233 (44.3) | 11478 (50.3) | 8756 (38.3) |
| >29 | 1237 (2.7) | 889 (3.9) | 348 (1.5) |
|  |  |  |  |
| Marital Status |  |  |  |
| Never married | 4369 (9.6) | 2781 (12.2) | 1588 (6.9) |
| Married | 38274 (83.8) | 18423 (80.7) | 19851 (86.8) |
| Widow/Separated/Divorced | 3042 (6.7) | 1619 (7.1) | 1422 (6.2) |
|  |  |  |  |
| Maternal education level |  |  |  |
| No formal education | 12618 (27.6) | 4374 (19.2) | 8244 (36.1) |
| Primary | 12811 (28.0) | 4933 (21.6) | 7878 (34.5) |
| Secondary | 16978 (37.2) | 10874 (47.6) | 6104 (26.7) |
| Post secondary | 3277 (7.17) | 2642 (11.6) | 635 (2.8) |
|  |  |  |  |
| Maternal occupation |  |  |  |
| Not working | 17463 (38.3) | 9452 (41.5) | 8011 (35.1) |
| Working | 28173 (61.7) | 13337 (58.5) | 14836 (64.9) |
|  |  |  |  |
| Wealth index |  |  |  |
| Poorest | 7481 (16.4) | 1160 (5.1) | 6320 (27.7) |
| .Poorer | 8780 (19.2) | 2454 (10.8) | 6325 (27.7) |
| Middle | 9328 (20.4) | 3979 (17.4) | 5349 (23.4) |
| Richer | 10204 (22.3) | 6592 (28.9) | 3613 (15.8) |
| Richest | 9891 (21.7) | 8638 (37.9) | 1253 (5.5) |
|  |  |  |  |
| Parity |  |  |  |
| ≥5 | 10684 (23.3) | 4483 (19.6) | 6200 (27.1) |
| 3-4 | 14552 (31.9) | 7548 (33.1) | 7004 (30.6) |
| 1-2 | 20448 (44.7) | 10792 (47.3) | 9657 (42.2) |
|  |  |  |  |
| ANC |  |  |  |
| <8 visits | 35672 (91.3) | 16820 (88.2) | 18852 (94.7) |
| 8+ visits | 3305 (8.5) | 2248 (11.8) | 1057 (5.3) |
| Birth interval |  |  |  |
| <33 months | 4575 (77.1) | 2487 (73.8) | 2087 (81.4) |
| 33+ months | 1361 (22.9) | 884 (26.2) | 477 (18.6) |
|  |  |  |  |
| Child’s size at birth |  |  |  |
| Very large | 4969 (10.9) | 2682 (11.8) | 2287 (10.0) |
| Larger than average | 10074 (22.1) | 4855 (21.3) | 5219 (22.8) |
| Average | 24547 (53.7) | 12099 (53.0) | 12449 (54.5) |
| Smaller than average | 4021 (8.8) | 2118 (9.3) | 1903 (8.3) |
| Very small | 1959 (4.3) | 1027 (4.5) | 932 (4.1) |
| Don’t know | 114 (0.25) | 43 (0.2) | 72 (0.31) |
|  |  |  |  |
| Cooking Fuel |  |  |  |
| Clean | 16275 (35.6) | 13829 (60.6) | 2446 (10.7) |
| Pollutant | 29409 (64.4) | 8994 (39.4) | 20415 (89.3) |
